# "APP"reciating the complexity of HIV-induced neurodegenerative diseases

**DOI:** 10.1371/journal.ppat.1007309

**Published:** 2018-10-25

**Authors:** Mojgan H. Naghavi

**Affiliations:** Department of Microbiology-Immunology, Northwestern University Feinberg School of Medicine, Chicago, Illinois, United States of America; Mount Sinai School of Medicine, UNITED STATES

Since the beginning of the epidemic in the 1980s, human immunodeficiency virus type 1 (HIV-1) has infected more than 70 million and killed approximately 35 million people globally. Although widespread use of combination antiretroviral therapy (cART) has effectively increased the life span of many infected individuals in the Western world, HIV-1 continues to be a major public health issue in both developed and resource-poor settings [1]. In addition to causing acquired immunodeficiency syndrome (AIDS), HIV-1 infection also causes serious neuropathology. Like all other retroviruses, upon entry into the host cell, HIV-1 converts its single-stranded RNA genome into double-stranded DNA that integrates into the host genome. To produce new viral particles or virions, HIV-1 proteins—together with the viral RNA—assemble at the plasma membrane, where a new immature virus forms. Virions then mature through processing of precursor group-specific antigen (Gag) polyprotein by HIV-1 protease at or after budding from the cell surface [2].

## Effects of HIV-1 infection on the central nervous system

**Fig 1:**
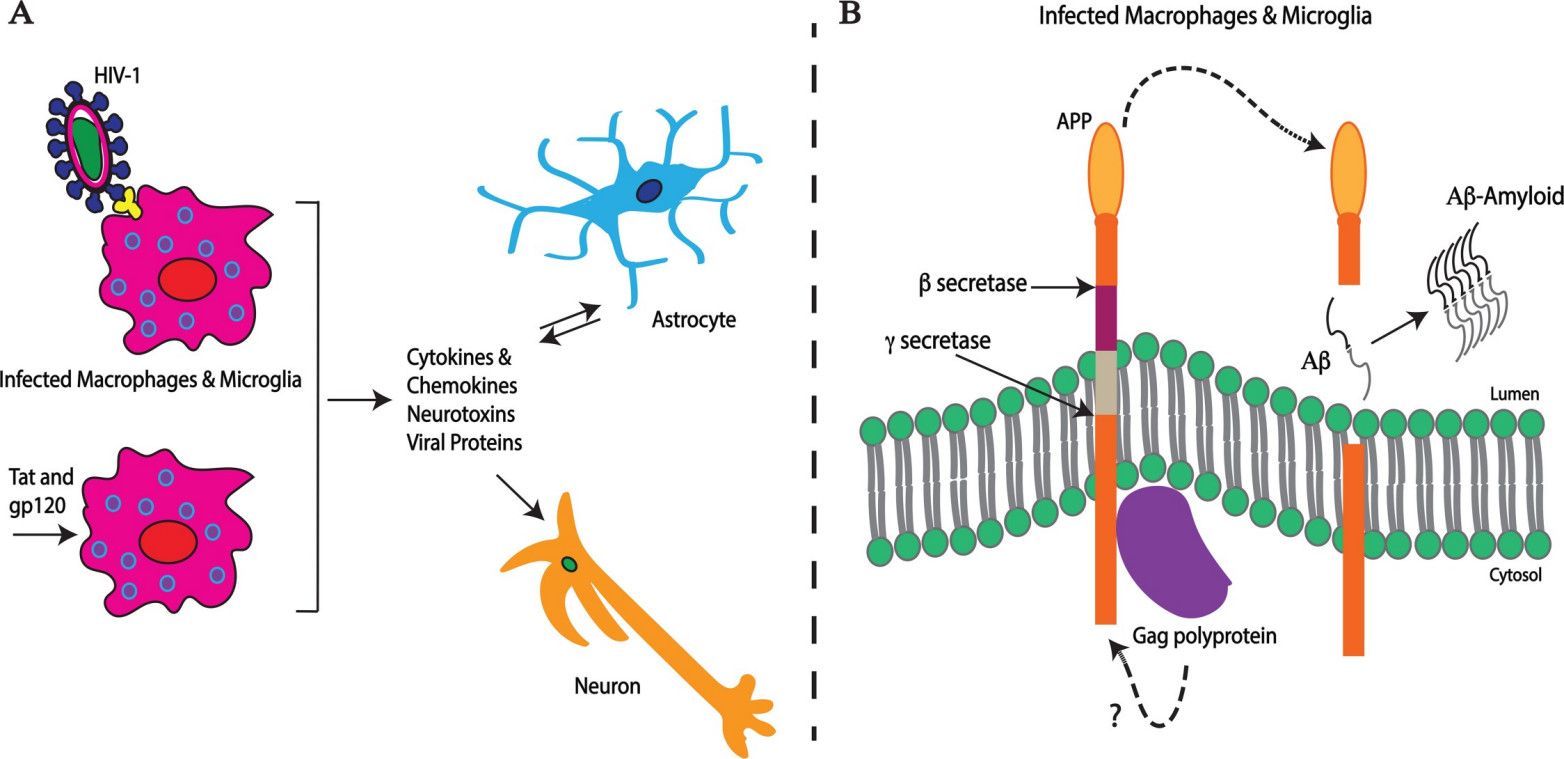
HIV-1 infection in the brain results in neurodegeneration. (A) Effects of HIV-1 on target cells in the CNS. Microglia and macrophages infected with HIV-1 and/or exposed to viral proteins (such as Tat and gp120) along with infected astrocytes secrete a mix of host and viral factors. This induces a toxic and inflammatory environment that contributes to the complex process of neuronal dysfunction and death in infected individuals that develop HAND. (B) HIV-1 Gag counteracts an innate restriction imposed by APP resulting in overproduction of neurotoxic Aβ. APP traps HIV-1 Gag polyprotein in cell membrane regions enriched in secretases during late-stage viral assembly and/or production in microglia or macrophages. To counteract this, Gag promotes secretase-dependent APP processing resulting in overproduction of toxic Aβ isoforms. Aβ, β-amyloid; APP, amyloid precursor protein; CNS, central nervous system; Gag, group-specific antigen; gp120, envelope glycoprotein 120; HAND, HIV-1 associated neurocognitive disorders; HIV-1, human immunodeficiency virus type 1; Tat, transactivator of transcription.

In many infected individuals, HIV-1 enters the central nervous system (CNS) leading to a broad spectrum of HIV-1–associated neurocognitive disorders (HANDs) ranging from mild impairments to severe HIV-associated dementia (HAD) [1]. Although HAD is rare in patients on cART, milder forms of HAND are still prevalent in these patients, likely due to irreversible injury prior to starting therapy, the persistence of virus in the CNS, and toxic effects of therapy itself [1, 3]. To enter the CNS, HIV-1 must cross the blood-brain barrier (BBB). This is believed to occur through a “Trojan horse” mechanism as a passenger in CD4+ cells, e.g., T cells or monocytes [4]. Indeed, HIV-1–mediated disruption of the integrity and function of the BBB plays an important role in AIDS neuropathogenesis [5]. Inside the CNS, HIV-1 establishes infection primarily in perivascular macrophages and microglia (Fig 1A) based on animal studies (in nonhuman primates or humanized mice) or acquired material during autopsy of HIV-1–positive individuals [4]. HIV-1 is also proposed to infect astrocytes to cause neuropathology, although this remains less well understood. Cell-free mature HIV-1 does not efficiently infect astrocytes but other routes of transmission, e.g., cell-to-cell transmission of immature HIV-1 virions from lymphocytes or engulfment of HIV-1–infected macrophage material has been linked to productive infection as well as functional changes during nonproductive infection of astrocytes that contribute to HAD [6, 7]. Because HIV-1 does not directly infect neurons, secretion of a mixture of host (inflammatory cytokines and chemokines) and viral proteins from these brain-resident infected cells is believed to induce localized inflammation resulting in HIV-1–induced neuronal dysfunction and death [4]. Neurotoxic HIV-1 proteins include envelope glycoprotein (Gp120), transactivator of transcription (Tat), the accessory viral protein R (Vpr), negative regulatory factor (Nef), and matrix p17 [8–12]. Beyond the toxic effects of viral proteins, it is clear that infected macrophages also secrete host factors that contribute to the broader neurodegeneration caused by HIV-1. Although these viral and host factors are too numerous and complex to discuss here and are extensively reviewed elsewhere [4], one poorly understood, yet potentially significant, host contributor is β-amyloid (Aβ).

## Aβ production and its role in neurodegeneration

Aβ is a small peptide that is generated throughout life but whose precise physiological function and reason for production remains somewhat mysterious. In fact, the peptide is best known as the main component of amyloid plaques that are associated with neurodegenerative conditions such as Alzheimer disease (AD) and dementia in uninfected individuals [13]. Neurotoxic Aβ is generated by site-specific cleavage of the amyloid precursor protein (APP). Neurons lacking APP exhibit impairments in neurite growth, intracellular trafficking, and cell-to-cell adhesion, suggesting that the precursor has important yet poorly understood biological functions in the brain. Although highly expressed in neurons, APP is a ubiquitously expressed type I membrane protein that is proteolytically processed near and within its membrane domain by four types of secretases (α, β, γ, and η) via three major pathways (amyloidogenic, nonamyloidogenic, and η-secretase) [14, 15] (Fig 1B). Most APP is sequentially cleaved by α- and γ-secretases producing a large N-terminal soluble fragment (sAPPα) in the extracellular space along with a short C-terminal fragment (α-CTF) and nontoxic peptide (p3) released into the cytoplasm via the nonamyloidogenic pathway. Less frequently, in the amyloidogenic pathway, APP is cleaved by β-secretase to generate a soluble ectodomain (sAPPβ) and a β-CTF. The latter is further processed by γ-secretase into Aβ monomers of various lengths that can self-associate to form toxic Aβ oligomers. There are two main toxic Aβ species, Aβ40 and Aβ42, although the smaller fraction Aβ42 is more prone to aggregation. Although the contribution of Aβ species to AD has been long debated, likely due to the complexity of its role, data suggest it is a promising target for AD therapeutics.

## The question of Aβ accumulation in HIV-1–infected individuals

Although Aβ increases in the brain during normal aging [16], pathology studies have shown that both APP and Aβ accumulation are accelerated in the brains of HIV-1–infected individuals, correlating with viral loads and the onset of HAND [17–19]. Postmortem tissue staining has also shown amyloid accumulation within the brains of simian immunodeficiency virus (SIV)-infected macaques and rats exposed to HIV-1 proteins [20, 21]. However, the question of Aβ deposition in infected individuals is complex and far from certain. In vivo positron emission tomography (PET) amyloid imaging has not detected increased Aβ42 fibrillar deposition in individuals with HAND [22]. However, this may reflect limitations of imaging or unusual features of HIV-1 Aβ deposition, discussed below. Elevated Aβ42 has also been reported in the cerebrospinal fluid (CSF) of HIV-1–positive patients, whereas others report no change or even decreases [23–27,28]. These kinds of conflicting observations are also evident in studies of AD and could be, among other concerns, due to the collection and handling of samples, an issue extensively reviewed elsewhere [29]. Similarly conflicting are reports of CSF levels of apolipoprotein E4 (APOE4), whose expression promotes amyloid aggregation and which is a well-established risk factor for AD [25, 30, 31]. Moreover, APOE4 appears to be more relevant in older HIV-1–positive patients [30]. Despite these uncertainties, which are also prevalent in AD research, soluble Aβ42 and APP in CSF have been suggested by many studies to serve as strong biomarkers for HAND [23, 24, 32, 33]. Moreover, suppressing Aβ42 accumulation has been proposed as a means for therapeutic intervention [34, 35]. Notably, intraneuronal Aβ accumulation or perivascular diffuse Aβ depositions are more prevalent in HIV-1–infected brains, whereas extracellular amyloid plaques are predominant features in AD [29, 36]. These distinct differences observed in Aβ deposition patterns between HAND and AD may explain the failure of PET imaging studies to detect fibrils (discussed above) and suggest that HIV-1 specifically alters Aβ metabolism in ways that may contribute to unique features of HAD and HAND. Indeed, soluble amyloid oligomers have been suggested as the primary pathological structures leading to permeabilization of cellular membranes and neuronal loss observed in AD [37].

## Effects of HIV-1 on APP and Aβ production

HIV-1 is known to alter multiple steps in Aβ metabolism [29, 36]. Several HIV-1 proteins such as Tat, gp120, Vpr, Nef, and the fusion Gag-polymerase polyprotein (Gag-Pol), have been shown to affect APP or Aβ levels, processing, aggregation, and/or subcellular localization [8, 10, 11, 38]. Although some of these viral proteins likely affect Aβ metabolism indirectly, other viral factors appear to function more directly. For example, gp120 and Nef can cause infected microglia or astrocytes, respectively, to release pro-inflammatory cytokines that increase Aβ42, whereas Tat can directly modulate APP trafficking and processing via inhibition of the major Aβ-degrading enzyme, neprilysin [29, 39, 40]. Tat is also known to reduce clearance of Aβ42 from the brain to the blood and promotes nuclear entry of Aβ and inflammatory responses in human brain endothelial cells [36]. However, Tat is secretory and is highly toxic, meaning that some of its effects on Aβ production may be indirect [4].

Recent findings have identified both a viral protein that induces Aβ production and biological reason for why this occurs. Although APP has few known biological functions, it was recently found to act as an innate restriction to HIV-1 release from brain-resident microglia and macrophages. To evade this restriction, the HIV-1 Gag polyprotein binds and directs secretase-driven processing and degradation of APP, resulting in Aβ production that can cause neurotoxicity (Fig 1B) [41]. Supporting this notion, another recent report also suggested a role for β-secretase BACE1 and Aβ oligomer production in HIV-associated neurotoxicity [42]. Because of this, Aβ production is at least in part a detrimental side effect of a viral evasion mechanism to escape a restriction to late-stage infection that is imposed by APP but that results in neurotoxic effects. Moreover, the virus-specific activities of Gag-induced APP processing along with the effects of other viral proteins on broader Aβ metabolism likely contribute to the underlying differences in Aβ deposition patterns in the brains of patients with HAND versus AD, discussed above. gp120 has been shown to increase APP levels [43], whereas APP can protect against gp120-induced brain damage [44]. In addition, Aβ has been proposed to potentially have antimicrobial activities [45]. It is therefore tempting to speculate that these earlier reports reflect the recent discovery of APP’s role in host responses to HIV-1 infection. Combined with inflammatory responses to infection and Tat cytotoxicity, Gag-mediated APP processing and Aβ production are likely to be an important contributing factor to the overall complexity of HAND. Furthermore, Gag-mediated induction of APP processing does not require the protease activity of the Gag-Pol fusion polyprotein that was previously reported to cleave APP [38]. Instead, Gag binds APP and enhances its processing by host secretases [41]. Inhibiting either β- or γ-secretase suppresses Gag-mediated Aβ production and protects against neurotoxicity [41]. By protecting APP from degradation, secretase inhibitors also suppress HIV-1 release and replication in microglia and macrophages, suggesting that these inhibitors may have the potential to serve dual purposes of preventing both neurodegeneration and HIV-1 replication in brain-resident target cells [41]. Although the role of APP and amyloid in HAND remains complex and somewhat controversial—similar to the situation with AD—these recent findings provide additional evidence that infected macrophage and/or microglia produce toxic amyloids and explain, at least in part, why and how infection causes this induction. However, there is undoubtedly much more to learn about the molecular basis by which this occurs, as well as the true extent to which Aβ contributes to neurodegenerative disease in infected patients.
